# Analysis of primitive genetic interactions for the design of a genetic signal differentiator

**DOI:** 10.1093/synbio/ysz015

**Published:** 2019-06-27

**Authors:** Wolfgang Halter, Richard M Murray, Frank Allgöwer

**Affiliations:** 1 Institute for Systems Theory and Automatic Control, University of Stuttgart, Stuttgart, Germany; 2 Division of Biology and Biological Engineering, California Institute of Technology, Pasadena, CA, USA; 3 Department of Control and Dynamical Systems, California Institute of Technology, Pasadena, CA, USA

**Keywords:** genetic circuit design, combinatorial promoters, signal differentiator

## Abstract

We study the dynamic and static input–output behavior of several primitive genetic interactions and their effect on the performance of a genetic signal differentiator. In a simplified design, several requirements for the linearity and time-scales of processes like transcription, translation and competitive promoter binding were introduced. By experimentally probing simple genetic constructs in a cell-free experimental environment and fitting semi-mechanistic models to these data, we show that some of these requirements can be verified, while others are only met with reservations in certain operational regimes. Analyzing the linearized model of the resulting genetic network, we conclude that it approximates a differentiator with relative degree one. Taking also the discovered nonlinearities into account and using a describing function approach, we further determine the particular frequency and amplitude ranges where the genetic differentiator can be expected to behave as such.

## 1. Introduction

The systematic design of functional genetic circuits is one of the key challenges in the field of synthetic biology. Usually, the goal is to add a desired function to a cellular organism. As the complexity of these functions has been increasing steadily (1), it becomes increasingly difficult to design the topology of the genetic network and decide what kind of genetic interactions to use. One way to approach this synthesis problem is by adapting methods from the fields of systems and control theory (2), e.g. by starting with a description of the desired part as a linear transfer function, finding the necessary fundamental input/output (I/O) functions which realize this transfer function and then realizing the evolving network topology with primitive genetic interactions. The key to this approach is to determine how fundamental linear I/O functions like gain, integrator, sum and difference can be realized using only primitive genetic interactions such as transcription, translation, combinatorial promotors, post-transcriptional modification or pairwise interactions of DNA, mRNA or protein molecules.

This design workflow follows the ideas of (3), where the authors showed that any arbitrary *linear* input/output system can be realized exactly using only zeroth and first order biochemical reactions. We addressed the question of replacing the zeroth and first order biochemical reactions with general genetic interactions in (4). Therein, several requirements were introduced to conclude that the processes of transcription and translation can be interpreted as gain and integration, respectively, and that combinatorial promoters may be used to realize the difference of two concentrations. In (4), and also in this work, we use these results to design a genetic signal differentiator, i.e. a genetic part whose output indicates the temporal derivative of its input. Such a module would be of particular interest in context of a genetic proportional-integral-derivative controller that could be used to regulate production processes within a cell. While for this purpose the genetic realization of the more important integral feedback has been studied extensively (5–10), differential operators in a biological context have been investigated rather sporadically (11, 12) and have only recently moved into the focus of synthetic biology (13). In latter work, the authors introduce a differentiator module based on mechanisms borrowed from the *E. coli* chemotaxis regulatory network. This mechanism is based on active enzyme-like degradation and the assumption that this degradation operates at saturation of the enzyme. In contrast to the results of (13), the topology presented in (4) is not based on a known biological example but is derived from scratch, using an adjusted version of the general design framework of (3). This leads to a differentiator module of similar complexity but different assumptions and requirements which need to be guaranteed.

In this work, we combine control theoretic concepts, mathematical models and observations from experiments to verify and adapt the requirements introduced in (4). We find that, in cell-free extract, transcription can be considered as a PT1 element, i.e. a delayed gain, while translation indeed can be seen as an integrator. Further, we show that combinatorial promoters are not very well suited to realize the difference of two signals and that the dynamics are very much dependent on the operation conditions. Lastly, we study how not meeting the requirements affects the performance of the genetic signal differentiator and reveal the operating conditions under which the differentiator behaves as expected and where this is not the case.

In the following, we first introduce the desired signal differentiator, one possible topology to realize this part and the necessary requirements for primitive genetic interactions by recapitulating the results established in (4). After, we introduce mathematical models of protein synthesis as well as the cell-free experimental environment which is used to generate the experimental data. Subsequently, the requirements on time-scales and linear operation regimes of the processes of transcription and translation are verified by fitting the model to a series of experimental data and analyzing the resulting parameters, leading to transfer function representations of these two processes. Using another series of experiments, we determine the input–output steady-state map of a combinatorial promoter and discuss the limited capability of such promoters to realize the difference of two signals. Finally, the impact of the discovered discrepancies on the performance of the genetic differentiator is studied both in time and frequency domain, using a describing function approach for the latter.

## 2. Background

First, we briefly recapitulate the results from (4) before we analyze, verify and adjust the requirements we introduced therein.

In the field of control theory, one can study linear systems in two different domains. First, in the time domain, by looking at the states of a system and the temporal derivatives thereof which define a system of ordinary differential equations (ODEs). And second, in the frequency domain, by looking at transfer functions which are complex valued functions and describe how different frequency components of an input signal are modified by a system. These two domains are connected via the Laplace transformation and particularly the frequency domain is very useful for the design and analysis of linear systems. An ideal differentiator would be given by the transfer function G(s)=s with Laplace variable *s*. However, as is well known in the control community, an exact realization of such an ideal differentiator is not possible due to the lack of causality. For a system to be causal, its output must not depend on future values of the input signal. This is not the case for the differentiator. In case the system is given in form of a rational transfer function, i.e. $G(s)=\frac{N(s)}{D(s)}$, one can easily check for this property by examining the degrees of the polynomials *N*(*s*) and *D*(*s*): causality is given if the degree of *N*(*s*) is not bigger than the degree of *D*(*s*).

**Figure 1.**
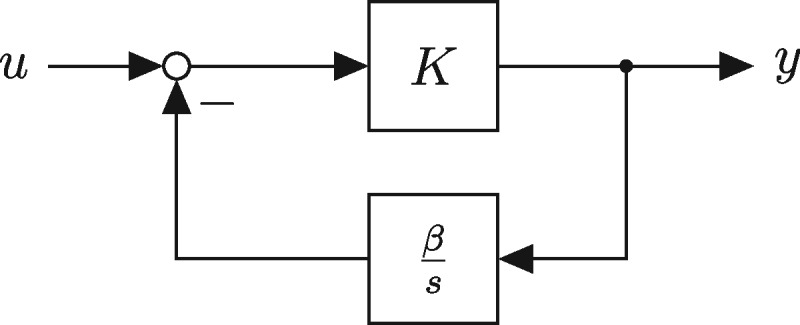
Ideal approximation of a differentiator, from (4).

The desired function thus can only be approximated, e.g. by adding an additional low-pass filter to the ideal differentiator, leading to the desired transfer function

(1) $$G(s)=\frac{\mathrm{Ks}}{s+K}$$

where *K* is the bandwidth of the filter. One possibility to realize this transfer function is by the circuit depicted in Figure 1, with a (preferably large) gain *K* in the forward path and a weighted integrator in the feedback path. Ideally, one chooses *β *= 1 to recover (1). Thus, in order to approximate the differentiator, three basic functions are needed: a gain, an integrator and the signal difference between input and feedback. Finding genetic realizations of these basic functions is the main challenge in designing the differentiator. In particular, it is expected that this cannot be achieved in an exact way, thus it is necessary to determine how inaccuracies in the basic parts influence the behavior of the assembled circuit. For an initial guess for finding such functions, a semi-mechanistic model of transcription and translation (14) was used in (4) to conclude that the processes of transcription and translation can approximately be seen as a gain and integrator, respectively, and that a combinatorial promoter may be used to realize the difference of two signals. In the remainder of this section, we briefly recapitulate these deductions.

In the process of protein synthesis, the genetic information is read from DNA (with concentration *D_i_*) and transcribed into mRNA (*M_i_*), then, mRNA molecules are translated into proteins (*P_i_*). In the following, the subscript *i* stands for the *i*th gene (*G_i_*) in a network with *I* distinct genes. With

$$P={\left[\begin{array}{ccc} P_{1} & \ldots & P_{I} \end{array}\right]}^{⊤}$$

representing all proteins present in the genetic network, the dynamics of mRNA and protein concentrations of gene *i* are described by

(2a) $$\dot{M_{i}}=f_{i}(P,\Theta_{i},\Phi )-p_{i}(M_{i},\Theta_{i},\Phi )$$

(2b) $$\dot{P_{i}}=g_{i}(M_{i},\Theta_{i},\Phi )-q_{i}(P_{i},\Theta_{i},\Phi )$$

where $f_{i}(P,\Theta_{i},\Phi )$ and $g_{i}(M_{i},\Theta_{i},\Phi )$ are the respective production and $p_{i}(M_{i},\Theta_{i},\Phi )$ and $q_{i}(P_{i},\Theta_{i},\Phi )$ the respective degradation rates. These rates are possibly dependent on protein and mRNA concentrations, certain gene-specific parameters $\Theta_{i}\in R^{N}$ like DNA concentrations (*D_i_*) or initiation and degradation rates, as well as several environmental parameters $\Phi \in R^{L}$ which include, among others, the total amount of RNA polymerase (RNAP), ribosomes and endonucleases, the transcription and translation elongation rates, and other host dependent variables. For better readability the arguments Θ_*i*_ and Φ are omitted in the remainder.

**Figure 2.**
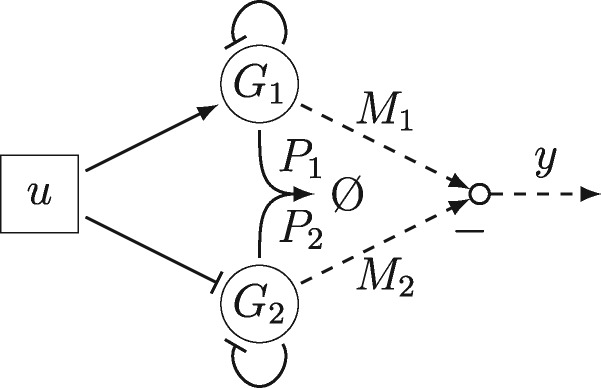
Genetic differentiator: Genes *G*_1_ and *G*_2_ tracking positive and negative slopes of *u*. Proteins produced by *G*_1_ and *G*_2_ neutralize each other. Difference of associated mRNAs indicate output *y*.

In (4), we introduced the topology depicted in Figure 2 as one approach to realize the transfer function (1). Therein, the input is considered to be a transcription factor, i.e. *u* = *P_u_*, which activates gene *G*_1_ and inhibits another gene *G*_2_. Each of these genes produces a transcription factor which suppresses its own production. While *G*_1_ has the purpose of capturing positive gradients of the input signal, *G*_2_ is designed to capture negative ones. The output of the part is then given as the difference between the mRNA concentrations of the two genes, i.e. $y=M_{1}-M_{2}$. Further, for the purpose of a minimal signal representation, the transcription factors *P*_1_ and *P*_2_ undergo an annihilation reaction.

Several simplifications and requirements for the processes of transcription, translation and degradation were introduced to finally arrive at the desired model equations

(3a) $${\dot{M}}_{1}=\alpha D_{1}\cdot \kappa (P_{u}-P_{1})-\nu_{1}M_{1}$$

(3b) $${\dot{P}}_{1}=\beta M_{1}-\delta_{1}P_{1}-\delta_{12}P_{1}P_{2}$$

(3c) $${\dot{M}}_{2}=\alpha D_{2}\cdot \kappa (k_{0}-P_{u}-P_{2})-\nu_{2}M_{2}$$

(3d) $${\dot{P}}_{2}=\beta M_{2}-\delta_{2}P_{2}-\delta_{12}P_{1}P_{2}$$

with the function

(4) $$\kappa (x)=\{\begin{array}{cc} x & x>0\\ 0 & x\leq 0 \end{array}$$

assuring strictly positive transcription rates. In the following, we focus on *G*_1_, the gene for capturing positive gradients, and recapitulate the requirements for the biological processes necessary to arrive at (3). Subsequently, the connection between (3) and (1) will be discussed. We note that the focus on *G*_1_ is without any loss of generality as the following requirements can be adjusted with minimal effort to arrive at the equations for *G*_2_.

**Requirement 1** *M*_1_ and *P*_1_ are subject to first order degradation, i.e.

(5a) $$p_{1}(M_{1})=\nu_{1}M_{1}$$

(5b) $$q_{1}(P_{1})=\delta_{1}P_{1}.$$

with degradation rate constants $\nu_{1},\delta_{1}\in \Theta_{1}$.

Although degradation rates *p_i_* and *q_i_* are usually dependent on protease and endonuclease levels, we require first order degradation dynamics to assure linearity with respect to mRNA and protein levels.

**Requirement 2** *The operation regime is such that f_1_ and g_1_ are both approximately linear in D_1_ and M_1_, respectively*.

This requirement is rectified by results like the ones presented in (15), where particularly the linearity of *g_i_* in *M_i_* is shown. Alternatively, similar simplifications have been applied by following a linearization approach as pursued in (16). In general, however, although the transcription rate *f_i_* increases monotonically with DNA concentration *D_i_*, it cannot grow arbitrarily large but is subject to saturation effects for large enough DNA or transcription factor concentrations, see e.g. (14, 17).

**Requirement 3** *There exists a combinatorial promoter which is piecewise linear in two inputs, such that*

$$f_{1}\left({\left[P_{u},P_{1}\right]}^{⊤}\right)∼\kappa \left(P_{u}-P_{1}\right)$$

*with* κ(·)*like in**Eq.* (4).

With this requirement, we demand that the combined effect of the two transcription factors is proportional to the difference of their concentrations, as long as $P_{u}>P_{1}$, and zero, otherwise. In other words, *f*_1_ as a function of ${\left[P_{u},P_{1}\right]}^{⊤}$, needs to fulfill the fundamental additivity property of linear functions in the regime $P_{u}>P_{1}$. This further means that, as we are considering a combinatorial promoter, *P_u_* has to act as an activator for *G*_1_ while *P*_1_ acts as an inhibitor. Consequently, instead of forming the difference between input *P_u_* and integral feedback *P*_1_ by using direct interactions between the two species, we move the difference operation to the promoter function.

Now if Requirements 2 and 3 hold, we find

(6a) $$f_{1}({\left[P_{u},P_{1}\right]}^{⊤})\approx \alpha D_{1}\cdot \kappa (P_{u}-P_{1})$$

(6b) $$g_{1}(M_{1})\approx \beta M_{1},$$

where *α* and *β* stand for lumped production rate parameters. Thus, with Requirements 1 to 3, we arrive at the first part of Eq. (3). Note that, when considering both genes *G*_1_ and *G*_2_, this means that the transcription and translation rate constants *α* and *β* are assumed to be equal for both genes. Also, it is required that $P_{u}>P_{1}$ for the part to work properly. For this reason, the annihilation reaction between *P*_1_ and *P*_2_ was introduced, see (4) for more details.

Finally, concerning an appropriate choice of parameters, another requirement can be deduced from typical degradation rates given e.g. in (18).

**Requirement 4** *The degradation of mRNA is much faster than the one of protein, i.e.* $\nu_{1}\gg \delta_{1}$.

With that in mind, one can apply a quasi steady state approximation of the mRNA dynamics and further assume that $\delta_{1}\approx 0$ to arrive at

$${\tilde{M}}_{1}\approx \frac{\alpha }{\nu_{1}}D_{1}(P_{u}-P_{1})$$

$${\dot{P}}_{1}\approx \beta {\tilde{M}}_{1}$$

where ${\tilde{M}}_{i}$ stands for the steady state mRNA concentration. Thus, we conclude that the process of transcription can be interpreted as a gain while translation approximately realizes an integrator. With the signal entering the transcription process chosen as the residual of input *P_u_* and integral feedback *P*_1_, the presented model thus realizes Eq. (1).

In (4), we verified this structure by simulating the system based on the much more detailed model described in (14). This detailed model mainly aims at taking the finite amounts of RNAP and ribosomes as well as the time delay of transcription and translation into account, however, chosen parameters only reflected average parameters from literature. Further, saturation effects and nonlinearities of the promoter dynamics were neglected.

After recapitulating the results of (4) and realizing the limitations of the used models, we now adjust our modeling approach and focus on analyzing and verifying the requirements by conducting a series of experiments using a cell-free experimental system (19).

## 3. Materials and methods

In this section, a brief overview on the experimental technique as well as the subsequently used models is provided.

### 3.1 TX-TL experimental platform

For the purpose of establishing a reliable, efficient and fast prototyping environment for genetic circuits, various cell-free TX-TL systems have been developed and optimized during the past decade (19–24). The main advantages of cell-free over classical cell-based *in vitro* systems are that cellular systems impose certain physical constraints on the gene circuits and the incorporation of the desired genes is comparably time consuming. Cell-free extracts on the other hand provide a well reproducible platform for rapid testing of arbitrary gene circuits. Such an extract for instance can be produced from *Escherichia coli* bacteria by bead-beating cell resuspensions, see (23) for more details on the production of *E. coli* extract. As DNA formatting and transformation as well as cell growth are thus decoupled from the actual testing of the circuit, testing cycles can be speed up significantly from several days for testing in original cells to only a few hours for testing in cell-free extract.

However, regeneration of resources required for mRNA and protein synthesis is an issue in cell-free environments, which is why the dynamics of mRNA and protein production are subject to some overlayed degradation dynamics of the extract. Therefore, the experiments are only meaningful for a limited experiment duration and we only consider observations within the first 200 min after initiation of the experiment. However, even in this limited time frame, degradation of resources will be visible in the experimental data. Since this mechanism is not considered in the mathematical models, the identified parameters will be biased. Production parameters tend to be underestimated while degradation parameters tend to be overestimated.

For every TX-TL experiment, the DNA subject to testing is suspended in water and mixed with cell extract and an energy buffer. This buffer contains amino acids, NTPs, tRNAs and other small molecules necessary for mRNA and protein synthesis. The reaction volume was chosen to 5μL. Usually, one or more genetic constructs encode a fluorescent reporter protein such as green fluorescent protein (GFP). After initialization of the experiment, the mixture is incubated at 29°C inside a Biotek plate reader, which assesses the level of fluorescent protein every few minutes. While the concentration of a fluorescent protein like GFP can be assessed directly, measuring the amount of mRNA requires an additional mechanism. We therefore make use of the malachite green dye (20 µM) and a corresponding aptamer sequence (MGapt) which is added to the 3ʹ untranslated region (UTR) of the gene. The dye binds to a binding pocket of this sequence and changes its emission properties upon binding, therefore again enabling us to monitor a fluorescence signal which is proportional to the mRNA concentration (25). However, measurements of the mRNA signal due to binding of the malachite green dye revealed only a poor signal to noise ratio, therefore an additional data pre-processing step was introduced by fitting a Gaussian process to the experimental data (26). Details on the pre-processing procedure can be found in Supplementary Data A.

In this work, we distinguish between gene- and extract-specific parameters. Gene-specific parameters include variables like the affinity of the particular promoter sequence toward RNAP and other proteins and by definition are considered to be independent of the environment the experiment is conducted in, i.e. hold in different batches of cell extract as well as inside living cells. In contrast, remaining parameters like the concentration of RNAP or transcription and translation elongation rates are denoted as extract or environment dependent, thus may vary even between different batches of cell-free extract. The experiments presented in this work have all been conducted using the same batch of TX-TL extract.

All genetic parts were originally given as plasmids. Using polymerase chain reactions and appropriate primer sequences, only the relevant linear double-stranded gene sequence was extracted from these plasmids and used in the TX-TL experiments. By addition of protein gamS, the degradation of linear DNA is prevented (27). Information about the used genetic constructs can be found in Supplementary Data B.

### 3.2 Modeling protein synthesis

Throughout this work, different promoters are discussed and analyzed for various purposes. Therefore, the different mechanisms and modeling framework used for simulating the temporal evolution of mRNA and proteins are introduced. We therein build upon the dynamics given in Equation (2), however, avoid using as strict simplifications as the ones outlined in Section 2.

In the following, complexes of two chemical species *A* and *B* are denoted with A:B and conserved quantities are indicated by a bar, e.g. $\bar{R}$, the total amount of RNAP.

It is a well-established result (18, 28) that the production rate of mRNA *f_i_* is proportional to the concentration of promoter which is bound to a corresponding RNAP holoenzyme and not blocked by any inhibitors, e.g.

(7) $$f_{i}(P)=\alpha \cdot D_{i}:R:\sigma_{70}(P,\Theta_{i},\Phi )$$

where the concentration of complex $D_{i}:R:\sigma_{70}$ may be depending on other proteins **P**, gene-specific parameters Θ_*i*_ and extract-specific parameters Φ.

In this example, sigma factor 70 (*σ*_70_) first has to bind to RNAP to form the holoenzyme before this complex then binds the promoter region. The sigma factor therein has a very high specificity toward certain promoters, enabling the cell to switch between different transcriptional programs depending on which sigma factor is expressed. Note that compared with (6a), this is a more realistic model for mRNA production but prohibits making the same deductions for the genetic differentiator.

The basic mechanisms of interest for us are binding and unbinding reactions happening at the promoter sequence of DNA. Usually, as in (18, 29), the amount of $D_{i}:R:\sigma_{70}$ is approximated by Michaelis-Menten like equations, assuming that either DNA or RNAP holoenzyme is in abundance. In contrast to that, we won’t make this assumption but particularly take the binding and unbinding reactions into account in order to consider both competition for shared cellular resources and saturation effects at the promoter. For simple setups where only self-competition occurs, we derive a closed form expression for the steady-state concentration of the respective biochemical complexes.

#### 3.2.1 Holoenzyme formation

When RNAP *R* is bound to a sigma factor *σ_x_*, this complex is referred to as the RNAP holoenzyme. As discussed briefly in the previous section, such a holoenzyme binds to the promoter sequence of a gene and initiates the transcription process. Therefore, sigma factors are a crucial component for this process and without the right sigma factor, transcription cannot initiate. According to (30), RNAP alone is sufficient for transcription elongation, however, initiation requires sigma factors. We therefore assume that the formation of holoenzyme is independent of the holoenzyme binding to the promoter sequence, meaning that sigma factor and RNAP can bind and unbind irrespective of the fact if RNAP is bound to DNA or not.

We therefore have to consider the reactions

(8a) $$R+\sigma_{x}\underset{k_{\sigma x}^{-}}{\overset{k_{\sigma x}^{+}}{⇌}}R:\sigma_{x}$$

(8b) $$D_{i}:R+\sigma_{x}\underset{k_{\sigma x}^{-}}{\overset{k_{\sigma x}^{+}}{⇌}}D_{i}:R:\sigma_{x}$$

for each sigma factor and DNA species present in the system in order to account for the competition for RNAP. To simplify (8), we introduce

$$\begin{array}{c} \bar{R:\sigma_{x}}=R:\sigma_{x}+\sum_{i}D_{i}:R:\sigma_{x}\\ X:R=R+\sum_{i}D_{i}:R \end{array}$$

the total amount of *R* bound to *σ_x_* as well as the total amount of *R* which is *not* bound to its respective sigma factor. Then, (8) can be combined to

(9) $$X:R+\sigma_{x}\underset{k_{\sigma x}^{-}}{\overset{k_{\sigma x}^{+}}{⇌}}\bar{R:\sigma_{x}}.$$

In most cases, only dissociation constants

$$K_{\sigma x}=\frac{k_{\sigma_{x}}^{-}}{k_{\sigma_{x}}^{+}}$$

are identifiable and it is assumed that binding reactions are fast compared with the transcription elongation steps and thus in quasi steady state. Therefore, for notational simplicity, we will reduce the notation to using dissociation constants instead of on and off rates in the remainder of this work.

Note that in (9) X:R and *σ_x_* denote both the unbound chemical species. If only one sigma factor is present in the system, the amount of $\bar{R:\sigma_{x}}$ can be calculated analytically as a function of the dissociation constant $K_{\sigma x}$ and the total amounts of RNAP and sigma factor, respectively, viz. by application of the following proposition.

**Proposition 1** Given the entities A, B and A:B and the reaction

$$A+B\underset{\overset{K}{⇌}}{}A:B.$$

*If none of the entities participates in any other chemical reaction, the steady state of* A:B*can be expressed in terms of the total amounts of A and B as*

(10) $$A:B=\frac{1}{2}\left(K+\bar{A}+\bar{B}-\sqrt{{\left(K+\bar{A}+\bar{B}\right)}^{2}-4\bar{A}\bar{B}}\right)$$

with $\bar{A}=A+A:B$ and $\bar{B}=B+A:B$.

The proof can be found in Supplementary Data C. It is noted that usually, i.e. for the deduction of Michaelis–Menten kinetics, it is assumed that either $\bar{A}\gg \bar{B}$ or $\bar{B}\gg \bar{A}$ holds while Proposition 1 gives exact solutions for any values of $\bar{A}$ and $\bar{B}$. In cases when only a single sigma factor is present and its total concentration is constant over the time course of the experiment, we will later on use the amount $\bar{R:\sigma_{x}}$ as a fitting parameter and omit the binding reaction in order to reduce the complexity of the fitting problem. However, in cases where the concentration of sigma factor varies over time, we either use the exact formula from Proposition 1, or if there is more than one sigma factor, we directly implement the binding reactions as fast reactions and accept the increased computational complexity.

#### 3.2.2 Promoter binding

After formation of $R:\sigma_{x}$, the RNAP holoenzyme binds to the promoter sequence and starts transcribing the information encoded as DNA. A promoter is called constitutive, if this binding of RNAP happens spontaneously and is not influenced by any activators or inhibitors, i.e.

(11) $$D_{i}+R:\sigma_{x}\underset{\overset{K_{\mathrm{iH}}}{⇌}}{}D_{i}:R:\sigma_{x}.$$

In such cases, given that the promoter does not interact with other holoenzymes, Proposition 1 can be applied again to simplify the modeling formalism.

In contrast to a constitutive promoter, binding of RNAP can also be inhibited by other proteins, leading to a combinatorial promoter with competitive binding mechanism, i.e. by the additional reaction

(12) $$D_{i}+P_{j}\underset{\overset{K_{\mathrm{ij}}}{⇌}}{}D_{i}:P_{j}$$

which now competes with (11).

#### 3.2.3 Translation and degradation rates

Similarly to the transcription rate (7), the rate of translation is given by

(13) $$g_{i}(M_{i})=\beta \cdot M_{i}:Q(M_{i},\Theta_{i},\Phi ),$$

where $M_{i}:Q$ stands for the concentration of ribosomes (*Q*) bound to the ribosome binding site of mRNA *M_i_*. We assume unregulated ribosomal binding and that the ribosome binding site sequences used for the constructs are of equal strength. Thus, the reactions for forming the complex $M_{i}:Q$ are the same as for the formation of holoenzyme and consequently, in case of only one mRNA species present, Proposition 1 can be applied again. Whenever more than one mRNA species is considered, competition for ribosomes occurs and binding reactions are implemented.

Degradation of mRNA and protein is mainly influenced by third party molecules such as endonucleases (*E*) and proteases. It is known (31) that latter species is quasi nonexistent in TX-TL extract, thus we keep the first order degradation for proteins as in (5b). Endonucleases, on the other hand, are present in limited quantities, thus loading effects need to be considered. We explicitly assume that the binding of ribosomes and endonucleases is independent of each other, i.e. can be seen as two distinct processes where ribosomes and endonucleases do not compete for mRNA. Thus, once more we define

(14) $$p_{i}(M_{i})=\gamma \cdot M_{i}:E(M_{i},\Theta_{i},\Phi )$$

and apply Proposition 1 whenever only self-competition occurs.

## 4. Results

Given the foundational work summarized in Section 2, it is yet unclear to what extent Requirements 1 to 4 can be verified. In particular, we are interested in answering the question of whether the processes of transcription and translation indeed can be regarded as a gain and integrator, respectively (Requirements 1, 2 and 4), and further, whether one can find a suitable combinatorial promotor which satisfies all linearity requirements in order to verify Requirements 3.

### 4.1 I/O behavior of transcription and translation

**Figure 3.**
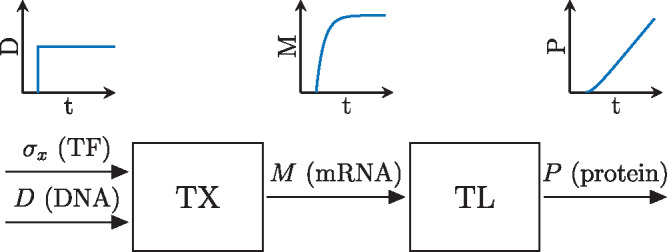
Scheme of probing protein synthesis with step in DNA and expected responses.

First, we analyze the time-scales and linearity of transcription and translation. Therefore, the I/O behavior of these processes are characterized by experimentally probing a simple gene with different input steps as depicted schematically in Figure 3. By observing the response to different step sizes in the input, the nonlinearity of the promoter dynamics can be identified. The gene we study is equipped with a *σ*_70_ dependent constitutive promoter and expresses GFP. By fitting a suitable model to the experimental data and analyzing the corresponding parameters, Requirements 1 and 4 will be verified.

#### 4.1.1 Experimental setup

There are two possibilities to realize a step-like input of varying height at the transcriptional level using promoters like introduced in Section 3.2: either by varying the amount of sigma factor (i.e. the transcription factor) while keeping the DNA concentration constant, or alternatively, changing the DNA concentration itself. While varying DNA amounts is straightforward, the sigma factor input additionally required purified protein which may be biologically unstable and is more difficult to obtain than DNA.

Depending on the choice of input, i.e. sigma factor or DNA, different dynamical effects can be expected when probing the system with steps of different height. As discussed before in Section 3.2, the mRNA production rate is proportional to the complex $D_{i}:R:\sigma_{70}$, wherein the concentration depends on the total amounts of DNA, RNAP and sigma factor. In case the concentration of sigma factor is considered as input, the corresponding model needs to incorporate both the formation of holoenzyme as well as the binding of holoenzyme to the DNA. Thus both binding rates would need to be considered. In contrast, when varying the DNA concentration, the binding reaction of holoenzyme can be neglected and the amount of total holoenzyme $\bar{R:\sigma_{70}}$ can be introduced instead.

This approach reduces the complexity of the fitting problem by focusing on the identification of promoter binding kinetics only. Thus, for the identification of the I/O behavior of transcription and translation, we first limit ourselves to step inputs in form of varying DNA concentrations and study the sigma factor dependent holoenzyme formation in a separate experiment, discussed in Section 4.2.

**Figure 4.**
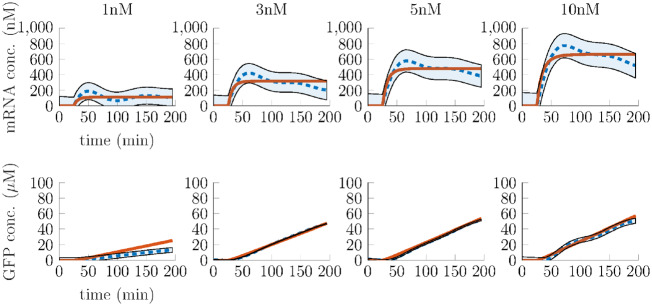
Mean and 95% confidence interval of experimental step responses (blue, dotted mean, shaded confidence interval) and simulated step responses of the fitted nonlinear model (red, solid).

We choose four different DNA concentrations for probing the system: 1, 3, 5 and 10 nM. Three technical replicates were conducted. The data obtained by this process are depicted in Figure 4. Therein, blue-dashed lines stand for the mean of mRNA (upper column) and protein (lower column) concentrations and the 95% confidence intervals are illustrated as shaded blue regions, respectively.

#### 4.1.2 Corresponding model

We denote the index of the gene under study with *i *=* *1 and accordingly the amount of GFP with *P*_1_. According to Section 3.2 and particularly Equations (2), (5b), (7), (13) and (14), the corresponding model is determined by the complexes

$$D_{1}:R:\sigma_{70}=D_{1}:R:\sigma_{70}\left({\bar{D}}_{1},\bar{R:\sigma_{70}},K_{1H}\right)$$

$$M_{1}:Q=M_{1}:Q\left({\bar{M}}_{1},\bar{Q},K_{\mathrm{MQ}}\right)$$

$$M_{1}:E=M_{1}:E\left({\bar{M}}_{1},\bar{E},K_{\mathrm{ME}}\right)$$

which are calculated using Proposition 1, depending on the total amounts of DNA, mRNA, RNAP holoenzyme, ribosomes and endonucleases as well as the respective dissociation constants. We again note that the model can capture the dynamics only in a limited time frame as the degradation of extract is not taken into account. For fitting the model to the given data, we introduce a maximum likelihood objective function, see e.g. (32), and apply several rounds of both patternsearch and fmincon optimization algorithms implemented in Matlab. The resulting parameters given in Table 1 give rise to the red trajectories depicted in Figure 4.

**Table 1.** Values of the parameters obtained by fitting the nonlinear model to step-response data

Parameter	Unit	Value	Description
α	min^−1^	21.54	Transcription rate const.
β	min^−1^	2.35	Translation rate const.
γ	min^−1^	0.18	mRNA deg. const.
δ	min^−1^	1.19e-8	Protein deg. const.
$K_{1H}$	nM	0.82	Dissoc. const. for $D_{1}$ and $R:\sigma_{70}$
$K_{\mathrm{MQ}}$	nM	72.26	Dissoc. const. for $M_{1}$ and Q
$K_{\mathrm{ME}}$	nM	102.2	Dissoc. const. for $M_{1}$ and E
$\bar{R:\sigma_{70}}$	nM	4.26	Total RNAP holoenzyme
$\bar{Q}$	nM	165.94	Total ribosomes
$\bar{E}$	nM	650.3	Total endonuclease

For the process of translation, we observe that the protein degradation rate *δ* is evaluated to be of magnitude $10^{-8}$ and therefore, compared with *γ*, practically zero.

**Figure 5.**
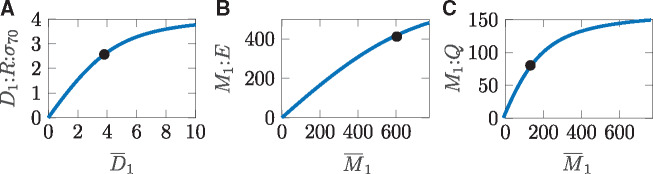
Amount of active complexes for transcription (**A**), mRNA degradation (**B**) and translation (**C**) over relevant range of DNA and mRNA, respectively.

**Conclusion 1** *As required in Requirements 4, the degradation of mRNA is much faster than the one of protein*.

In order to check the linearity Requirements 1 and 2, we study the entities $D_{1}:R:\sigma_{70},\;M_{1}:E$ and $M_{1}:Q$ as functions of the fitted parameters over the relevant range of DNA and mRNA concentrations as depicted in Figure 5. This way, one can visualize the nonlinear nature of the production reactions of mRNA and protein as well as the degradation of mRNA. Although these results clearly indicate that the processes of transcription and translation do not behave linearly in their inputs in general, they allow us to define operation regimes as those required in Requirements 2, i.e. where the linearity requirement holds at least approximately.

In that sense, we now introduce a relative measure of nonlinearity and define the *ϵ*-linear-range of a function f:R→R as the largest interval $\left[0,\xi^{*}\right]$ for which this nonlinearity measure is just *ϵ*. For the nonlinearity measure, we follow the methods introduced in (33). Let $||f(x){\|}_{L_{2}[0,\xi ]}$ be the truncated L2 norm of *f*(*x*), defined by

$$||f(x){\|}_{L_{2}[0,\xi ]}=\sqrt{\frac{1}{\xi }\int_{0}^{\xi }f{\left(x\right)}^{2}dx}.$$

To approximate *f*, we use the linear function *mx*. Note that we forced the intercept of the linear function to take the value 0 to assure strictly positive values of the linear function on the interval [0,ξ]. For a given *f*, the best linear approximation in the interval [0,ξ] is then found as the argument $m=m^{*}$ which minimizes

(15) $$L(\xi ,m)=||(f(x)-\mathrm{mx}){\|}_{L_{2}[0,\xi ]},$$

the absolute L2 norm of the residual between function *f*(*x*) and the linear function *mx*. The value of $L(\xi ,m^{*})$ now can be seen as an absolute measure for the nonlinearity of *f* on the interval [0,ξ], however, this measure depends on the magnitude of the function *f*. Thus, in order to compare this measure across different functions, we normalize (15) by the L2 norm of *f*, i.e.

$$L_{\text{rel}}(\xi ,m)=\frac{||(f(x)-\mathrm{mx}){\|}_{L_{2}[0,\xi ]}}{||f(x){\|}_{L_{2}[0,\xi ]}}$$

to find our relative measure of nonlinearity.

Consequently, $\xi^{*}$ is found as the solution of

(16) $$\begin{array}{cc} \max\; & \xi \\ s.t.\; & \underset{m}{\min}L_{\text{rel}}(\xi ,m)\leq \epsilon . \end{array}$$

In the given case, when one allows for a 5% error, i.e. ϵ=0.05, one obtains the linear ranges indicated as black points in Figure 5.

**Conclusion 2** *Linearity of production and degradation terms, as requested in Requirements 1 and 2, can be verified with 95% accuracy with*

$$\begin{array}{cc} D_{1}:R:\sigma_{70}\approx A_{\text{tx}}\cdot \bar{D_{1}} & \mathrm{for}\;\;\;\;\bar{D_{1}}\in [0,3.805]\\ M_{1}:E\approx A_{\text{deg}}\cdot \bar{M_{1}} & \mathrm{for}\;\;\;\bar{M_{1}}\in [0,593.1]\\ M_{1}:Q\approx A_{\text{tl}}\cdot \bar{M_{1}} & \mathrm{for}\;\;\;\;\bar{M_{1}}\in [0,141.7] \end{array}$$

and $A_{\text{tx}}=0.726,\;A_{\text{deg}}=0.752,\;A_{\text{tl}}=0.602$.

#### 4.1.3 Linearized model and transfer functions

Given the linear operation regimes indicated in Conclusion 2, one can now derive linear models for transcription and translation which are then valid in the respective regimes. In the control community, the standard approach to approximate a nonlinear model with a linear one is to locally linearize the nonlinear function at on specific value. In case of the nonlinear mRNA degradation rate *p*_1_ for example, a linearization around some fixed value ${\bar{M}}_{1}^{0}$ would yield

$$p_{1}({\bar{M}}_{1})\approx p_{1}({\bar{M}}_{1}^{0})+{\frac{dp_{1}}{d{\bar{M}}_{1}}|}_{{\bar{M}}_{1}^{0}}\cdot ({\bar{M}}_{1}-{\bar{M}}_{1}^{0}).$$

This approach assures that the linear function evaluated at ${\bar{M}}_{1}^{0}$ has the same value as the original nonlinear one, and that the difference between the two functions is small in a neighborhood around ${\bar{M}}_{1}^{0}$. Thus, the quality of the linear model on a certain interval strongly depends on the chosen value ${\bar{M}}_{1}^{0}$. In our case, particularly the values of ${\bar{M}}_{1}$ may vary across a wide range. Further, it should be made sure that in the case when neither DNA nor mRNA or protein is present, the temporal derivatives of these species also is equal to zero, i.e. that

$$\dot{{\bar{M}}_{1}}({\bar{D}}_{1}=0,{\bar{M}}_{1}=0)=\dot{{\bar{P}}_{1}}({\bar{M}}_{1}=0,{\bar{P}}_{1}=0)=0$$

holds. This will only be achieved if all linear functions go through the origin. To assure this, one would consequently have to perform the linearization at ${\bar{D}}_{1}={\bar{M}}_{1}={\bar{P}}_{1}=0$, leading to potentially large deviations between the linear and nonlinear models at larger values of the independent variables. Therefore, instead of using this standard approach, we directly use the approximations of Conclusion 2 where we already made sure that the linear approximation is as good as possible over a given interval of the independent variable.

We thus obtain the linear model

$$\begin{array}{c} \dot{{\bar{M}}_{1}}\approx \alpha A_{\text{tx}}{\bar{D}}_{1}-\gamma A_{\text{deg}}{\bar{M}}_{1}\\ \dot{{\bar{P}}_{1}}\approx \beta A_{\text{tl}}{\bar{M}}_{1}-\delta {\bar{P}}_{1} \end{array}$$

and when defining ${\bar{D}}_{1}$ and ${\bar{M}}_{1}$ as input and output of the transcription module, ${\bar{M}}_{1}$ and ${\bar{P}}_{1}$ as input and output of the translation module, the corresponding transfer functions

(17) $$G_{\text{tx}}(s)=\frac{\alpha A_{\text{tx}}}{s+\gamma A_{\text{deg}}}$$

(18) $$G_{\text{tl}}(s)=\frac{\beta A_{\text{tl}}}{s+\delta }$$

are obtained. We conclude that due to the fact that *δ* is very small, translation can indeed be seen as integration as long as DNA and mRNA concentrations are in the appropriate operation regime. However, the initial assumption that transcription can be seen as a gain needs to be adjusted as mRNA degradation cannot be neglected, leading to a PT1 element instead of a gain.

So far, we studied and characterized time-scales and linearity of the processes of transcription and translation in context of an *E. coli* cell-free extract and mainly focused on possible limitations caused by the promoter and mRNA-binding kinetics. We therefore bypassed nonlinear effects of RNAP holoenzyme formation by changing DNA concentrations instead of using *σ*_70_ as input and found that at least during the first 200 min of a TX-TL experiment, resource limitations do have an effect on transcription, translation and mRNA degradation. By studying different step responses, the linear operation regimes were identified. We now turn toward inhibitor binding dynamics and in particular toward the problem of how to realize a signal difference using combinatorial promoters.

### 4.2 Signal difference and combinatorial promoters

In order to approximate the derivative of a signal by implementing the scheme depicted in Figure 1, we remember that the input into the gain (i.e. transcription) has to be the residual between the reference and feedback signal.

There are various ways to realize a signal difference in biology, a widely used one being sequestration-based mechanisms between the signaling molecules, e.g. binding and degradation of the complex like elaborated in (7, 8). When dealing with RNA or DNA, such a mechanism can be realized in a straightforward way by e.g. the use of antisense strands. When it comes to proteins or metabolites, engineering a sequestration mechanism for an arbitrary protein or metabolite may be possible but in general is more challenging. Thus, one is rather restricted to the use of existing pairs of proteins which undergo binding reactions, e.g. sigma factors and antisigma factors. Combinatorial promoters as an alternative mechanism may offer a higher flexibility during the prototyping process as various inhibitor operator sequences are already known for transcriptional regulation. Therefore, it is in principle possible to compare the concentrations of any two transcription factors by combination of these operator sequences with different promoters. It is one of the goals of this work to investigate whether this approach can actually be used for the purpose of subtraction in a biological context.

**Figure 6.**
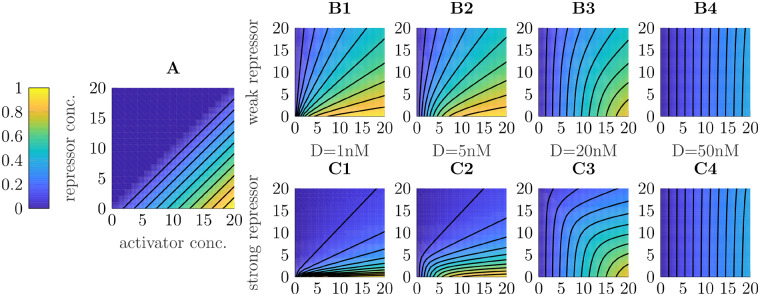
Level sets of promoter activity $D_{i}:R:\sigma_{x}$ over varying levels of sigma factor and inhibitor. (**A**) Desired behavior for non-negative signal difference. (**B1**–**B4**) Simulated values for varying DNA concentrations under a weak repressor. (**C1**–**C4**) Simulated values for varying DNA concentrations under a strong repressor.

Following such an approach, the desired behavior of the steady state of promoter dynamics is depicted in Figure 6A where the steady state of $D_{i}:R:\sigma_{x}$ is color-coded over varying concentrations of ($\bar{\sigma_{x}}$) and inhibitor (${\bar{P}}_{j}$). Due to non-negativity of concentrations, no activity is desired whenever the concentration of inhibitor exceeds the one of activator (upper left triangle resembling zero). Otherwise, it is aspired that $D_{i}:R:\sigma_{x}$ is proportional to the difference $\bar{\sigma_{x}}-{\bar{P}}_{j}$, illustrated by the parallel and equidistant level sets in Figure 6A.

Applying Proposition 1 and assuming that the total amount of RNAP holoenzyme is fixed, the amount of $D_{i}:R:\sigma_{x}$ depends on the chosen DNA concentration as well as on dissociation constants *K_iH_* and *K_ij_* of the RNAP holoenzyme and inhibitor, respectively. If for instance we assume that $K_{\mathrm{iH}}=K_{\mathrm{ij}}=1$ and look at the relative amount of activated DNA $D_{i}:R:\sigma_{x}/{\bar{D}}_{i}$, varying holoenzyme and inhibitor in the same range results in qualitatively different steady-state maps depending on how much ${\bar{D}}_{i}$ is chosen, as depicted in Figure 6B1–B4. For high DNA, sigma factor acts quasi linearly on the promoter while the inhibitor does not play a role at all. On the other hand, for small amounts of DNA, the inhibitor has a large effect and distorts the steady-state map such that the level sets converge to each other at the origin. Also, suppression due to the repressor does not seem strong enough as in all cases, $D_{i}:R:\sigma_{x}\gg 0$ for $\sigma_{x}<P_{j}$.

In contrast to that, Figure 6C1–C4 show the same conditions, except that now $K_{\mathrm{iH}}=10\cdot K_{\mathrm{ij}}$, i.e. the inhibitor binds 10 times stronger to the promoter than RNAP holoenzyme does. In that case, only minimal transcriptional activity is expected when there is less sigma factor than repressor. Further, although level sets are curved, for medium amounts of DNA, e.g. 20 nM, they are comparably equidistant and the steady-state map is almost symmetric.

This means that, while we have to acknowledge that exact realization of the difference of two signals is not possible with combinatorial promoters, some crucial properties can be approximated by choosing dissociation constants and DNA amounts carefully.

For that purpose and also for detangling the RNAP holoenzyme binding reaction, we study a gene with a *pTar* initiation sequence combined with a *tetO* inhibitor operator which expresses GFP. The *pTar* promoter is sensitive toward an RNAP holoenzyme consisting of RNAP bound to *σ*_28_, while the operator sequence *tetO* enables binding and inhibition through Tet repressor proteins (tetR). We denote the concentration of this gene as *D*_2_ and GFP concentration as *P*_2_.

To avoid usage of purified protein, both *σ*_28_ and *tetR* are produced in the TX-TL system from respective constitutive (i.e. *σ*_70_ dependent) DNAs $D_{s28}$ and *D_tetR_*. While the amount of ${\bar{D}}_{s28}$ is varied to achieve different activation levels, inhibition is influenced by adding different amounts of anhydrotetracycline (*aTc*) which binds to *tetR* and thus alleviates its association with the promoter. The concentration of ${\bar{D}}_{\mathrm{tetR}}$ is kept at a constant level of 1 nM. The combinatorial promoter then produces GFP, dependent on the concentrations of *σ*_28_ and unblocked *tetR*. The time-series of this experiment can be found in Supplementary Data D.

According to the experimental setup, several chemical species compete for the same resources, thus Proposition 1 cannot be applied anymore and the binding reactions themselves had to be implemented as fast reactions. For brevity reasons, the binding reactions are not listed here. We focus on mRNA and protein dynamics, i.e. the ODEs

$$\begin{array}{c} {\dot{\bar{M}}}_{s28}=\alpha \cdot D_{s28}:R:\sigma_{70}-\gamma \cdot M_{s28}:E\\ {\dot{\bar{\sigma }}}_{s28}=\beta \cdot M_{s28}:Q-\delta \cdot {\bar{\sigma }}_{s28}\\ {\dot{\bar{M}}}_{\mathrm{tetR}}=\alpha \cdot D_{\mathrm{tetR}}:R:\sigma_{70}-\gamma \cdot M_{\mathrm{tetR}}:E\\ \dot{\bar{\mathrm{tetR}}}=\beta \cdot M_{\mathrm{tetR}}:Q-\delta \cdot \bar{\mathrm{tetR}}\\ {\dot{\bar{M}}}_{2}=\alpha \cdot D_{2}:R:\sigma_{28}-\gamma \cdot M_{2}:E\\ {\dot{P}}_{2}=\beta \cdot M_{2}:Q-\delta \cdot P_{2}. \end{array}$$

Fitting these equations to the data, we obtain the parameters listed in Table 2 and the trajectories depicted in Supplementary Data D. In the fitting process, the optimization is constrained such that the amount of complex $\bar{R:\sigma_{70}}$ is similar to the value fitted in the first experiment where binding of sigma factor has been neglected, see Table 1.

**Table 2.** Values of the parameters obtained by fitting the nonlinear model to the time-series responses of the combinatorial promoter

Parameter	Unit	Value	Description
$K_{\sigma 70}$	nM	1.8e–6	Dissoc. const. for R and $\sigma_{70}$
$K_{\sigma 28}$	nM	5.3e–3	Dissoc. const. for R and $\sigma_{28}$
$K_{\mathrm{tetR}}$	nM	8.1e–3	Dissoc. const. for$\;D_{2}$ and tetR
$K_{\mathrm{aTc}}$	nM	2.74	Dissoc. const. for tetR and aTc
$K_{2H}$	nM	1.084e4	Dissoc. const. for $D_{2}$ and $R:\sigma_{28}$
$K_{s28H}$	nM	33.86	Dissoc. const. for $D_{s28}$ and $R:\sigma_{70}$
$K_{\mathrm{tetRH}}$	nM	2.97e3	Dissoc. const. for $D_{\mathrm{tetR}}$ and $R:\sigma_{70}$
$\bar{R}$	nM	283.14	Total RNAP
${\bar{\sigma }}_{70}$	nM	3.36	Total sigma factor 70

The values given in Table 2 indicate that the total amount of RNAP is much bigger than the one of *σ*_70_ and further, that binding between these two species is very strong. Although *σ*_28_ also binds strongly to RNAP, its affinity is still smaller than the one of *σ*_70_. The excessive amount of RNAP and the much higher binding affinity of *σ*_70_ thus leads to a decoupling of the two binding reactions.

**Figure 7.**
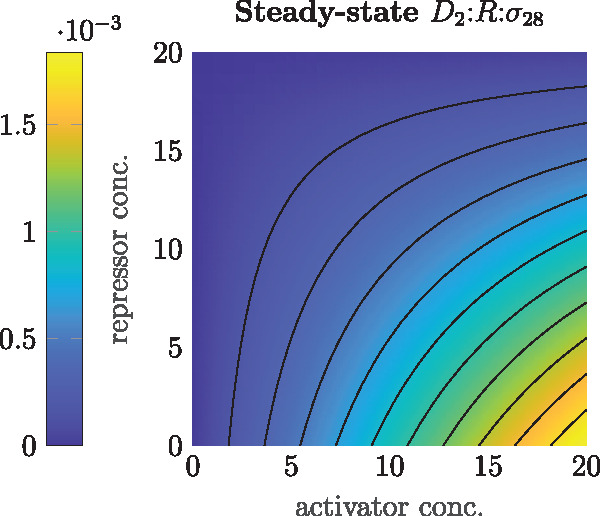
Promoter activity of *pTar-tetO*, obtained from fitted model.

We also note that the binding of $R:\sigma_{28}$ to the *pTar* promoter apparently has a very low affinity which leads to low GFP levels compared with the input step experiments. Together with the fact that the repressor *tetR* binds the *pTar* promoter very strongly, this leads to the steady-state promoter map depicted in Figure 7, where the amount of active promoter for 20 nM of DNA and varying activator and inhibitor concentrations is determined based on the reactions from Section 3.2 and parameters from Table 2.

Although there is leakage for medium amounts of inhibitor and activator and the level sets are not completely linear, the determined promoter dynamics are comparable to the desired behavior of Figure 6A.

**Conclusion 3** Using combinatorial promoters, the difference between two signals can only be realized to a limited extent.

**Figure 8.**
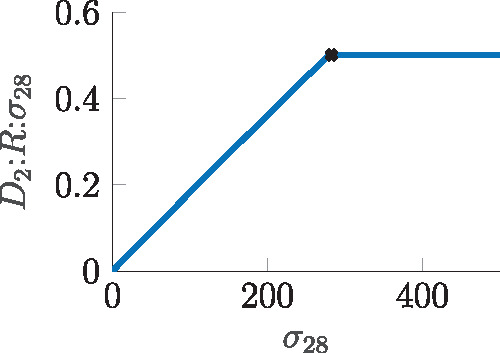
Amount of active transcription complex over relevant range of sigma factor concentration for *pTar* promoter.

Given these results the transcription dynamics of Section 4.1 can now be extended with the appropriate promoter dynamics and *σ*_28_ as input. As pointed out before, the strong binding affinities of the sigma factors lead to a quite linear but bi-modal I/O behavior, as depicted in Figure 8, compared with the one depicted in Figure 5A. Therein, the active $D_{2}:R:\sigma_{28}$ complex linearly follows the amount of *σ*_28_ until the concentration of RNAP is matched. Consequently, the transcriptional gain *A_tx_* changes due to the change of input and using the same linear approximation as defined in (16), one now finds

$$D_{2}:R:\sigma_{28}\approx {\tilde{A}}_{\mathrm{tx}}\cdot \sigma_{28}$$

(19) $$\text{with }{\tilde{A}}_{\mathrm{tx}}=0.0018.$$

### 4.3 Implications for the closed loop

Initially, with Requirements 1 to 4, we expected the process of transcription to behave like a gain, translation to behave like an integrator and combinatorial promoters to provide the difference of two signals. Now, several observations were made which differ from our initial view.

First, although mRNA degradation is indeed much faster than protein degradation, the simplification to a simple gain is not justified and the temporal dynamics of mRNA production should be taken into account instead, leading to a PT1 behavior instead of a gain.

Second, both production and degradation rates are subject to saturations due to finite amounts of resources of the transcriptional and translational machinery in the cell-free extract. For small inputs, however, these rates can be seen as linear functions of their inputs and the linear operation regimes have been determined explicitly.

Third, when realizing the difference of two signals by using combinatorial promoters, one only obtains an approximation of the difference and the quality of the estimate depends on the magnitudes of the inputs.

Now that these deviations from our initial requirements have been identified and characterized, their effect on functionality and performance of the synthetic genetic differentiator postulated in (4) can be studied. For that purpose, two different models are compared with the ideal realizable differentiator from Equation (1) in both time and frequency domain.

**Figure 9.**
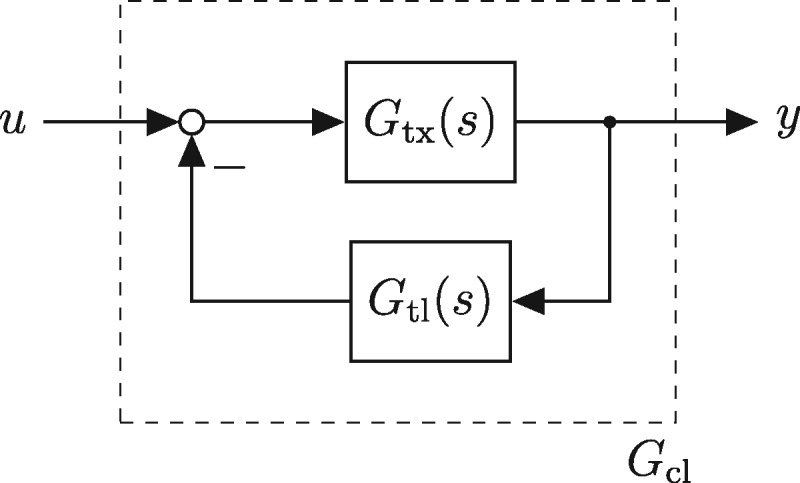
Topology of the linearized model given as the closed loop of $G_{\text{tx}}$ and $G_{\text{tl}}$.

The first model is given by the closed loop of the models $G_{\text{tx}}$ and $G_{\text{tl}}$ given in (17) and (18), respectively, and adapted with the new transcriptional gain (19). This results in a linear model like depicted in Figure 9 where no saturation effects are taken into account and perfect signal difference is assumed. However, the slow mRNA production and resulting PT1 behavior is taken into account and parameters of $G_{\text{tx}}$ and $G_{\text{tl}}$ resemble realistic values as they were obtained from experimental data. With the simplification *δ *= 0, the transfer function of the closed loop system thus is given by

(20) $$G_{\mathrm{cl}}(s)=\frac{\alpha {\tilde{A}}_{\text{tx}}s}{s^{2}+\gamma A_{\text{deg}}s+\alpha \beta {\tilde{A}}_{\text{tx}}A_{\text{tl}}}.$$

The second model is considered as the detailed nonlinear model and is based on the reactions introduced in Section 3.2, thus taking all saturation effects, nonlinearities and time-delays into account. It consists of a gene *G*_1_ with a combinatorial promoter like the one studied in Section 4.2, i.e. sensitive to a *σ*_28_ holoenzyme and *tetR* inhibitor, producing this very same inhibitor, therefore realizing the circuit from Figure 1. The concentration of *σ*_28_ is considered as input signal. In order to capture both positive and negative gradients, the same approach as introduced in (4) is used, leading to a network topology like in Figure 2 where *G*_2_ is of similar structure as *G*_1_ but with negative influence of the input on the transcription rate. The following additional mechanisms are necessary to realize this topology:

- Additionally to *σ*_28_, a second sigma factor *σ_xx_* is introduced to be present at a constant level. While $R:\sigma_{28}$ activates transcription of *G*_1_ and $R:\sigma_{\mathrm{xx}}$ activates the one of *G*_2_, both holoenzymes bind to both genes, leading to a competition and negative influence of one to the other.
- Self-inhibition of the two genes is achieved by two different inhibitors, e.g. *tetR* and $\mathrm{tet}R^{*}$.
- The two inhibitors *tetR* and $\mathrm{tet}R^{*}$ undergo an annihilation reaction at rate
- $\mu =0.1{\left(nM.\min\right)}^{-1}$ which was chosen arbitrarily.

As these modifications have been discussed in (4) already, we omit the details at this point. The mRNA and protein dynamics of the core species as well as the output of the system is given by

(21a) $${\dot{\bar{M}}}_{\mathrm{tetR}}=\alpha \cdot D_{\mathrm{tetR}}:R:\sigma_{28}-\gamma \cdot M_{\mathrm{tetR}}:E$$

(21b) $$\dot{\bar{\mathrm{tetR}}}=\beta \cdot M_{\mathrm{tetR}}:Q-\delta \cdot \bar{\mathrm{tetR}}-\mu \cdot \mathrm{tetR}\cdot \mathrm{tet}R^{*}$$

(21c) $${\dot{\bar{M}}}_{\mathrm{tet}R^{*}}=\alpha \cdot D_{\mathrm{tet}R^{*}}:R:\sigma_{\mathrm{xx}}-\gamma \cdot M_{\mathrm{tet}R^{*}}:E$$

(21d) $$\dot{\bar{\mathrm{tet}R^{*}}}=\beta \cdot M_{\mathrm{tet}R^{*}}:Q-\delta \cdot \bar{\mathrm{tet}R^{*}}-\mu \cdot \mathrm{tetR}\cdot \mathrm{tet}R^{*}$$

(21e) $$y={\bar{M}}_{\mathrm{tetR}}-{\bar{M}}_{\mathrm{tet}R^{*}}.$$

We summarized the core features of these two models and the desired circuit in Table 3. Note that Model 1 can be seen as the linearized version of Model 2.

**Table 3.** Summary of the models and comparison of the core features

	Desired circuit	Model 1	Model 2
Topology	Figure 1	Figure 9	Figure 2
Dynamics	Equation (1)	Equation (20)	Equation (21)
Features	Linear	Linear	Nonlinear
	Perfect gain	Delayed gain	Delayed gain
	No saturation	No saturation	Saturation
	Perfect difference	Perfect difference	Approximated difference

#### 4.3.1 Frequency domain analysis

**Figure 10.**
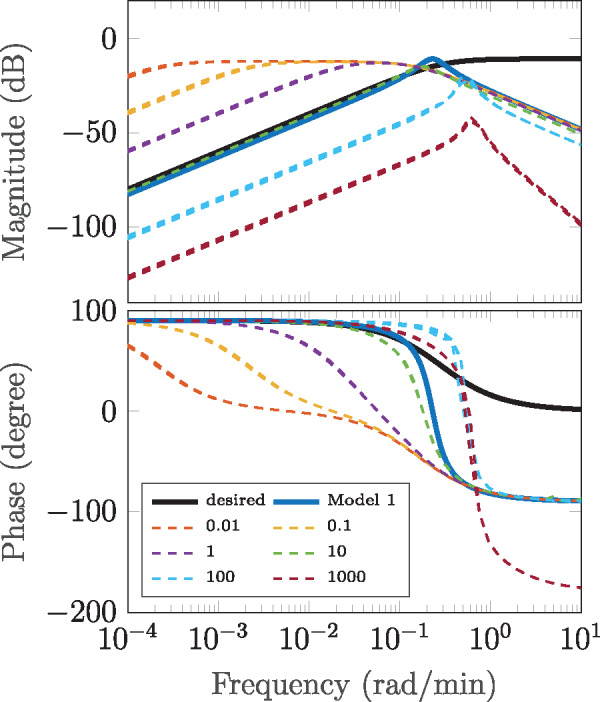
Bode plot of desired model (solid black) and linear Model 1 (solid cyan). In dashed lines, magnitude and phase of output of nonlinear Model 2 subject to $u(t)=A_{0}+A\cdot \;\text{sin}(\omega t)$. Different colors indicate different values of *A*_0_. Several values for *A* are plotted (lying on top of each other).

In a first step, we compare the two models and desired behavior in the frequency domain, i.e. in terms of the Bode plot depicted in Figure 10. This again is a classical tool from the control community and graphically shows how sinusoid input signals are modified by a certain transfer function. In the upper part, the magnitude amplification Λ(ω) indicates how the amplitude of the input signal is amplified for different input frequencies. In the lower part, the phase shift Ω(ω) for these frequencies is shown. Magnitude and phase of the desired behavior (solid black) and linearized model (solid blue) are obtained trivially using Matlab.

For the nonlinear model, however, we use a describing function approach as described in (34) to compare the I/O behavior of the nonlinear Model 2 with the linear ones. Therefore, the nonlinear Model 2 is excited with input

(22) $$u(t)=A_{0}+A\cdot \;\text{sin}(\omega t)$$

and the corresponding output *y*(*t*) analyzed in terms of its Fourier coefficients. Assume that after time $t^{*}=k^{*}\frac{2\pi }{\omega }$, the output oscillates in a steady-state fashion, i.e. no transient dynamics occur anymore, and let

$$c_{n}(\omega ):=\frac{1}{T}\int_{t^{*}}^{t^{*}+T}y(t)e^{-\mathrm{in}\omega t}dt\text{ with period}$$

$$T:=\frac{2\pi }{\omega }$$

be the *n*th Fourier coefficient of signal *y*(*t*) which corresponds to frequency *ω*. Then, the magnitude amplification Λ will be given as the ratio of the magnitudes of the first Fourier coefficients of output and input signal. With the input defined like in (22), the first Fourier coefficient of this signal is simply $\frac{A}{2i}$. Therefore, we have

$$\Lambda (\omega )=\frac{\left|c_{1}(\omega )\right|}{\left|\frac{A}{2i}\right|}=\frac{2|c_{1}(\omega )|}{A}.$$

Further, the phase shift Ω for this frequency and particular input signal is given by

$$\Omega (\omega )=\text{atan}\left(-\frac{\text{Re}c_{1}(\omega )}{\text{Im}c_{1}(\omega )}\right).$$

The constant part *A*_0_ of input signal (22) is necessary to produce non-negative sinusoid functions. Due to the nonlinearity of Model 2, the output *y*(*t*) does not only depend on the frequency *ω* but the shape of the input function in general, i.e. also the variables *A*_0_ and *A*. We therefore probed the system for several frequencies and values for *A*_0_ and *A*.

**Figure 11.**
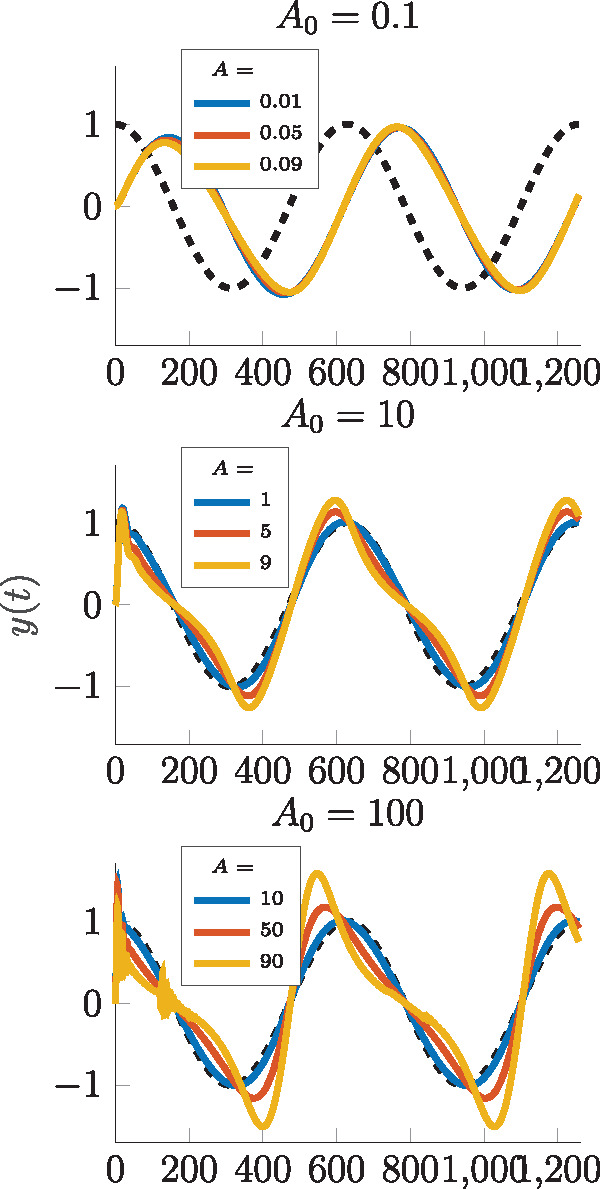
Normalized output $\tilde{y}(t)$ of the nonlinear closed model with input $u(t)=A_{0}+A\cdot \;\text{sin}(0.01t)$ for varying values of *A*_0_ and *A*.

For linear systems, an input signals with a single frequency component, like the one of (22), leads to an output with also only one frequency component, namely the same as the one of the input. In other words, higher harmonics are not existent and $|c_{n}(\omega )|=0$ for *n *>* *1. This is not the case for general nonlinear systems where higher harmonics can also appear and in principle more than just the first Fourier coefficient should be analyzed. Thus, the way we use the describing function approach in this work relies on the assumption that higher harmonics of the output signal can be neglected. We thus analyzed the power spectrum of the output signals for different values of *A*, *A*_0_ and *ω* and found that for most combinations, the higher harmonics contributed less than 5% to the overall power spectrum. However, in the case when *A* approaches *A*_0_ and *ω* is close to the pole of the transfer function, it seems that the assumption does not hold, see Supplementary Data E for further details. We will see in Section 4.3.2 and Figure 11 what this means for the output signal.

In Figure 10, magnitudes and phases of the respective response signal are plotted as dashed lines where different colors indicate different values for *A*_0_. The values for *A* are chosen as $A=kA_{0}$ with k∈[0.1,0.5,,0.75] and respective responses plotted in the same color. As seen in Figure 10, the output response does not change with varying *A*, however, the choice of the offset *A*_0_ significantly influences the I/O behavior of the nonlinear signal differentiator. Very low values of *A*_0_ (dashed red, orange and purple) lead to a very sensitive response, i.e. too high gain of the resulting closed loop and a smaller range of frequencies for which the output approximates the derivative of the input.

A value of $A_{0}=10$ (dashed green) results in the best response of the nonlinear system, matching the gain of an ideal differentiator quite well while providing almost the same frequency range as the one predicted by the linearized system ($\omega_{\text{max}}\approx 0.03$ rad/min). For too large values of *A*_0_ (dashed cyan and dark red), Model 2 breaks down as expected due to the previously characterized saturation effects and the resulting loss of sensitivity toward the input signal.

#### 4.3.2 Time domain analysis

From the previous analysis, we summarize that for the detailed Model 2, the phase of the output signal is off for too small values of *A*_0_, the gain is very small for values of $A_{0}\gg 10$ and only in case of $A_{0}\approx 10$ both magnitude and phase are as desired. We now focus on the shape of the output signal of Model 2 and therefore stick to sinusoid input signals, fixing ω=0.01 but varying *A*_0_ and amplitude *A* of the input signal. The normalized output

(23) $$\tilde{y}(t)=\frac{1}{\omega A}y(t)$$

as response to the just described input is depicted in Figure 11.

For a small value of *A*_0_, as expected, the phase is off, however, the output signal still has a sinusoid shape for all amplitudes *A*. In contrast, for higher values of *A*_0_, the phase is correct but with amplitude *A* approaching the offset *A*_0_, the output signal becomes more and more distorted. This effect is amplified for higher offset values and is caused by a dilution of the power spectrum as discussed in the previous section.

## 5. Discussion

For the synthesis of genetic networks that realize arbitrary linear transfer functions, we follow a similar approach as in (3). Therefore, it is crucial to find suitable genetic counterparts to primitive I/O functions such as gain, integration and difference. In a first attempt discussed in (4) and recapitulated in Section 2, several requirements were introduced to associate the processes of transcription and translation and combinatorial promoters with these respective I/O primitives. Now, a series of experiments and analyses was presented to verify and adapt these requirements.

By observing mRNA and protein levels as response to step inputs of varying height, it was verified in Conclusion 1 that protein degradation is almost nonexistent while mRNA degradation is comparably fast. However, degradation dynamics are not as fast as desired and a quasi steady state assumption for the process of transcription would be oversimplifying. Thus, transcription should be considered as a PT1-element rather than a gain.

By fitting an ODE model to the experimental data and analyzing the corresponding parameters, it was also shown that all processes are subject to saturation due to limited amounts of resources. Using the same model and the fitted parameters, the linear operation regimes of the I/O primitives can be characterized as shown in Conclusion 2, leading to more insight into the capabilities and limitations of respective genetic circuits.

In a second series of experiments, the dependence of the performance of a combinatorial promoter on the operation regime was emphasized, realizing in Conclusion 3 that the difference of two signals can only be obtained approximately. Based on these insights, DNA concentrations for a simulation study were chosen such that the I/O behavior of the combinatorial promoter is as close as possible to the desired one. In conclusion, the use of combinatorial promoters for comparing the concentrations of two transcription factors is only possible within a limited range of magnitudes and we suggest to use sequestration-based mechanisms in future.

For the realization of a genetic signal differentiator using the studied parts, the initial goal was to realize a differentiator with high-pass filter. The corresponding transfer function is given in Equation (1). It has a zero at the origin and one pole determined by the filter to make it a causal system. However, slow mRNA degradation leads to a behavior which, when linearized, is of relative degree one, i.e. Equation (20) which has one zero at the origin and two poles in the left half plane. This reveals an additional delay of the transient dynamics.

If protein degradation were significantly larger than zero, this would lead to a transfer function of the form

(24) $$G_{\mathrm{cl},\mathrm{protdeg}}=\frac{K_{1}s+K_{1}\gamma_{2}}{s^{2}+(\gamma_{1}+\gamma_{2})s+\gamma_{1}\gamma_{2}+K_{1}K_{2}},$$

thus, shifting the zero from the origin to the right half plane and therefore leading to an additional lower frequency bound and a sign change in the output. In comparison, the differentiator introduced in (13) leads to a very similar transfer function as (24), given that all necessary assumptions introduced there hold. The main difference is that in (13), the zero of the transfer function always lies in the left half plane. On one hand, this means that a sign change is avoided. On the other hand, there inherently exists a lower bound for admissible input frequencies while for the design presented in this work, this only is be the case if protein degradation is large.

In order to conduct studies beyond the linearized model, a describing function approach is used to evaluate the response of the nonlinear model to sinusoid inputs like in Equation (22). Therein, it can be seen that the performance of the differentiator critically depends on the constant part of the input signal, revealing again the limitations due to resource competition but also unexpectedly toward some supersensitivity at low values of *A*_0_. With an appropriate choice of *A*_0_, the presented network approximates the temporal derivative of an input signal for frequencies up to ω≈0.02 rad/min. Additionally to the dependence on the absolute value of *A*_0_, simulations in the time domain revealed a dependence on the relative amplitude $\frac{A}{A_{0}}$ in sense of a distortion of the output signal. When this relative amplitude approaches the value 1, the output signal looses its similarity to the sinusoid input, although phase and gain may be correct. In other words, the nonlinearities of the model lead to a dilution of the power spectrum of the output and higher harmonics are amplified.

## Supplementary Material

ysz015_Supplementary_Data
