# Supplementary material for: Analysis of primitive genetic interactions for the design of a genetic signal differentiator
Source: Synth Biol (Oxf). 2019 Jun 27;4(1):ysz015. doi: 10.1093/synbio/ysz015 (PMC7445770; doi:10.1093/synbio/ysz015)
Supplement: ysz015_Supplementary_Data [file ysz015_supplementary_data.pdf]

Analysis of primitive genetic interactions for the design of a genetic signal differentiator.

Wolfgang Halter[1,*] Richard M. Murray[2] Frank Allgöwer[1]

[1] Institute for Systems Theory and Automatic Control, University of Stuttgart, 70569 Stuttgart, Germany and [2] California Institute of Technology, Pasadena, CA 91125, USA

*Corresponding author: E-mail: wolfgang.halter@ist.uni-stuttgart.de

Supplementary Data

A Data pre-processing

In this section we denote the data obtained in the experiments discussed in Sections 4.1 and 4.2 with $y \in \mathbb{R}$. There are mainly three issues with these data, exemplarily depicted in Fig. 12 **A** and **B** as grey crosses.

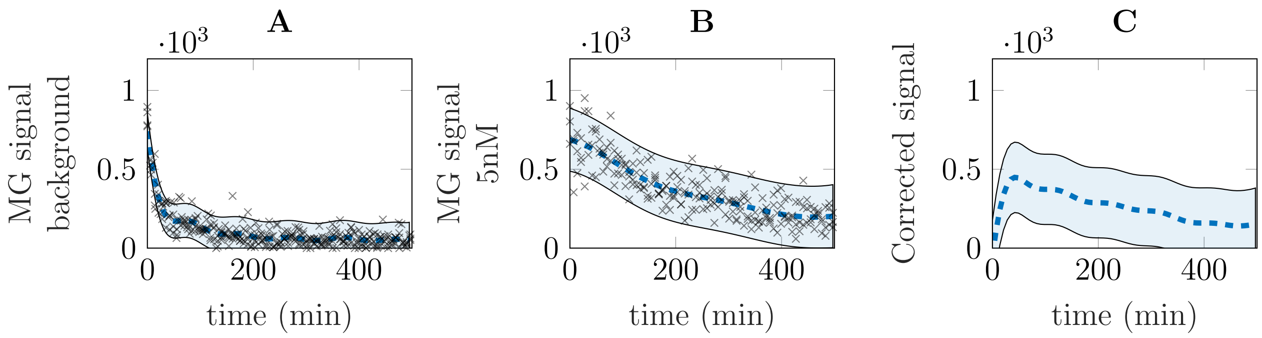

Figure 12: Data pre-processing of I/O experiments. Example: processing malachite green signal with 5nM of DNA.

First, the measurements are corrupted with noise, i.e.

$$y = f(t) + \rho,\ \rho \sim \mathcal{N}(0, \epsilon)$$

where $f(t)$ is some deterministic process generating the noise-free data and ρ the gaussian noise. This is particularly the case for the malachite green fluorescence measurements. Second, the time points at which the measurements are obtained are not uniformly spaced due to inconsistent preparation times of the experiments. This leads to a heterogeneous distribution of the measurements along the time axis. And last, for malachite green, a substantial part of the measured signal stems from some background signal caused by unbound malachite green, leading to the need of correcting the signals by subtracting the background part. However, due to the non-uniform temporal spacing of the measurements, a correction of the background requires some kind of model or interpolation scheme of the data.

We therefore assume that the measurement noise ρ is i.i.d. and model the timeseries for each experimental condition as a gaussian process, i.e.

$$y \sim \mathcal{GP}\left(\mu, k(t, t', \theta) + \epsilon^2 \delta_{tt'}\right)$$

where $\mu \in \mathbb{R}$ is a constant mean, k is chosen as a squared exponential kernel parametrized with θ and $\delta_{tt'}$ being the Kronecker delta.

Now let $y^{(ctrl)}$ and $y^{(e)}$ be the fitted gaussian processes of a control experiment without any DNA and some other experimental condition with predicted mean $\mu_\star^{(ctrl)}$, $\mu_\star^{(e)}$ and predicted standard deviations $\sigma_\star^{(ctrl)}$, $\sigma_\star^{(e)}$ as derived in [34] and depicted in Fig. 12 as dashed blue lines (mean) and light blue shaded area (standard deviation). The background corrected signal $\tilde{y}^{(e)}$ is then determined by

$$\tilde{\mu}_\star^{(e)} = \mu_\star^{(e)} - \mu_\star^{(ctrl)}$$
$$\left(\tilde{\sigma}_\star^{(e)}\right)^2 = \left(\sigma_\star^{(e)}\right)^2 + \left(\sigma_\star^{(ctrl)}\right)^2,$$

like depicted in Fig. 12 **C**.

Finally, the fluorescence signals are converted from the arbitrary intensity unit into a concen-

tration unit, using the previously obtained calibration relations

$$1723\, a.u. = 1\mu M \text{ GFP}$$
$$775\, a.u. = 1\mu M \text{ mRNA.}$$

B Genetic constructs

Gene	functional contents	sequence information
D_1	pBest-deGFP-MGapt	addgene.org/67734/
D_2	pTar-tetO-deGFP	see * for sequence
D_{s28}	pBest-$\sigma_2$8	addgene.org/45779/
D_{tetR}	pBest-tetR	addgene.org/45778/

*GGCATGCCAAGCTTCAATAAAGTTTCCCCCCTCCTTGCCGATAATCCCTATC
AGTGATAGAGAGCTAGCAATAATTTTGTTTAACTTTAAGAAGGAGATATACCA
TGGAGCTTTTCACTGGCGTTGTTCCCATCCTGGTCGAGCTGGACGGCGACGTA
AACGGCCACAAGTTCAGCGTGTCCGGCGAGGGCGAGGGCGATGCCACCTACGG
CAAGCTGACCCTGAAGTTCATCTGCACCACCGGCAAGCTGCCCGTGCCCTGGC
CCACCCTCGTGACCACCCTGACCTACGGCGTGCAGTGCTTCAGCCGCTACCCC
GACCACATGAAGCAGCACGACTTCTTCAAGTCCGCCATGCCCGAAGGCTACGT
CCAGGAGCGCACCATCTTCTTCAAGGACGACGGCAACTACAAGACCCGCGCCG
AGGTGAAGTTCGAGGGCGACACCCTGGTGAACCGCATCGAGCTGAAGGGCATC
GACTTCAAGGAGGACGGCAACATCCTGGGGCACAAGCTGGAGTACAACTACAA
CAGCCACAACGTCTATATCATGGCCGACAAGCAGAAGAACGGCATCAAGGTGA
ACTTCAAGATCCGCCACAACATCGAGGACGGCAGCGTGCAGCTCGCCGACCAC
TACCAGCAGAACACCCCCATCGGCGACGGCCCCGTGCTGCTGCCCGACAACCA
CTACCTGAGCACCCAGTCCGCCCTGAGCAAAGACCCCAACGAGAAGCGCGATC
ACATGGTCCTGCTGGAGTTCGTGACCGCCGCCGGGATCAGAAGGGAAGAAAGA
GCAAAGAAGGTAGCATAA

C Models

This section extends the results presented in Section 3.2.

Proof of Proposition 1. By setting $\frac{d}{dt}A = 0$, one arrives at the quadratic equation

$$(A{:}B)^2 - A{:}B(K + \overline{A} + \overline{B}) + \overline{A}\overline{B} = 0$$

which in general can have either none, exactly one or two real solutions, determined by the discriminant

$$\Delta = (K + \overline{A} + \overline{B})^2 - 4\overline{A}\overline{B}.$$

Taking into account that only $K > 0$, $\overline{A} > 0$ and $\overline{B} > 0$ are biologically meaningful, one finds

$$\Delta = K^2 + K\overline{A} + K\overline{B} + (\overline{A} - \overline{B})^2 \geq 0$$

thus at least one real solution exists. For the existence of exactly one solution, one would need $K = 0$ which was excluded previously. Otherwise, the quadratic formula yields

$$A{:}B_{1,2} = \frac{1}{2}\left(K + \overline{A} + \overline{B} \pm \sqrt{(K + \overline{A} + \overline{B})^2 - 4\overline{A}\overline{B}}\right)$$

where we assign $A{:}B_1$ to the solution with the negative and $A{:}B_2$ to the one with the positive sign. Due to mass conservation, we are interested in the solution for which

$$0 \leq A{:}B_i \leq \min(\{\overline{A}, \overline{B}\}) \tag{25}$$

holds. Now, $0 \leq A{:}B_i$ for both $i = [1, 2]$ follows directly from

$$\left(K + \overline{A} + \overline{B}\right)^2 \geq \left(K + \overline{A} + \overline{B}\right)^2 - 4\overline{A}\overline{B}.$$

and with

$$\frac{1}{2}\left(K+\overline{A}+\overline{B}\right) \geq \frac{1}{2}\left(K+2\min(\{\overline{A},\overline{B}\})\right)$$
$$> \min(\{\overline{A},\overline{B}\})$$

it can be seen that $A{:}B_2$ violates (25). It remains to realize that

$$K+\overline{A}+\overline{B}-2\min(\{\overline{A},\overline{B}\}) \leq$$
$$\sqrt{(K+\overline{A}+\overline{B})^2-4\overline{A}\overline{B}}$$

to conclude that $A{:}B_1$ is the only biologically meaningful solution. □

D pTar promoter characterization

The time series data of the pTar characterization experiment is depicted in Fig. 13.

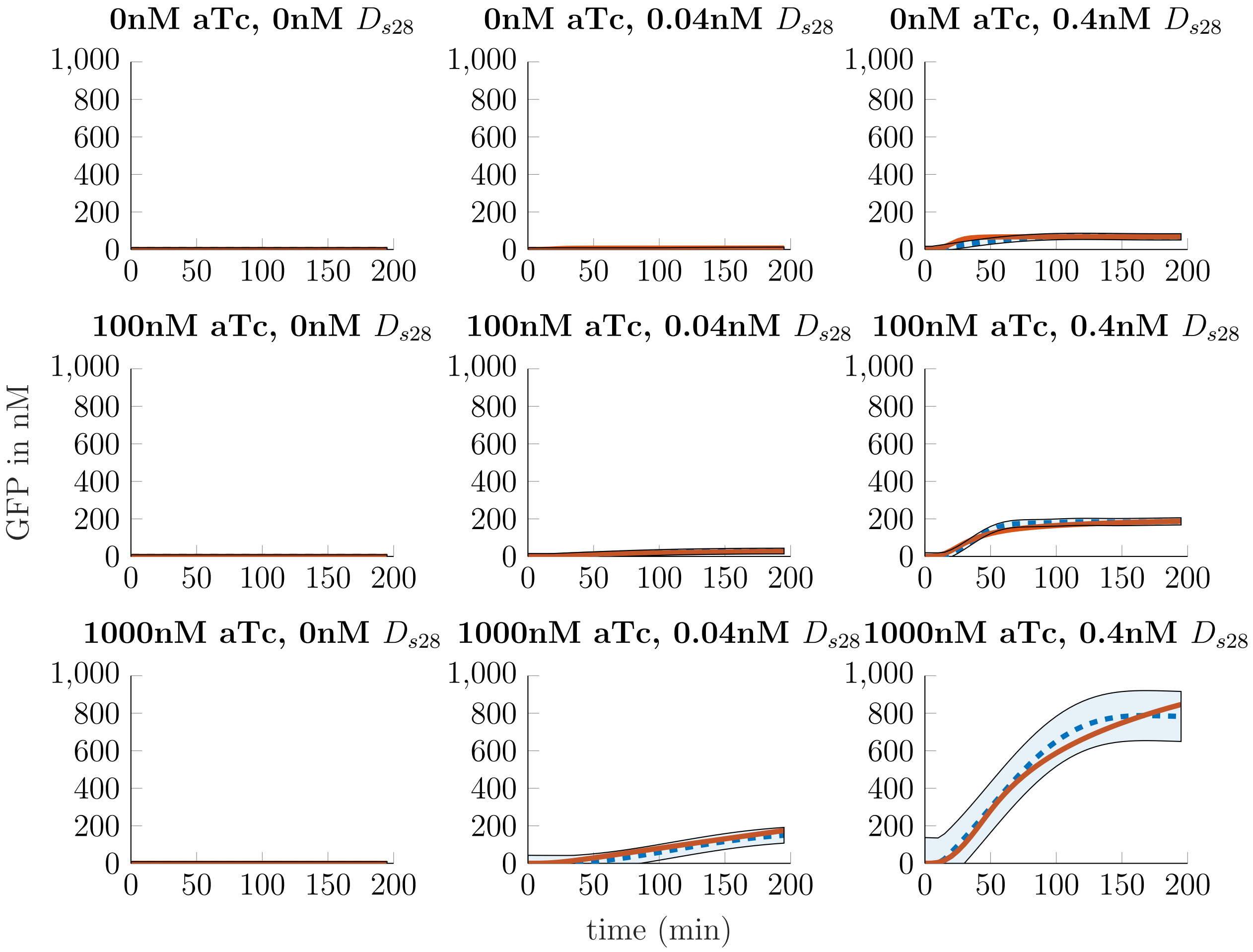

Figure 13: Concentration of GFP over time. pTar concentration at 5 nM, varying sigma factor DNA $\overline{D}_3$(increasing from left to right) and inhibitor concentrations (increasing from top to bottom).

E Limitations of the Describing Function approach

The way the Describing Function approach has been used in Section 4.3.1, we assume that higher harmonics can be neglected in the output signal. This, however, may not the be case for every combination of parameters A, A_0 and ω of the input signal given in (22). Therefore, we analyzed the output signal of the nonlinear Model 2 in terms of its 10 first Fourier coefficients

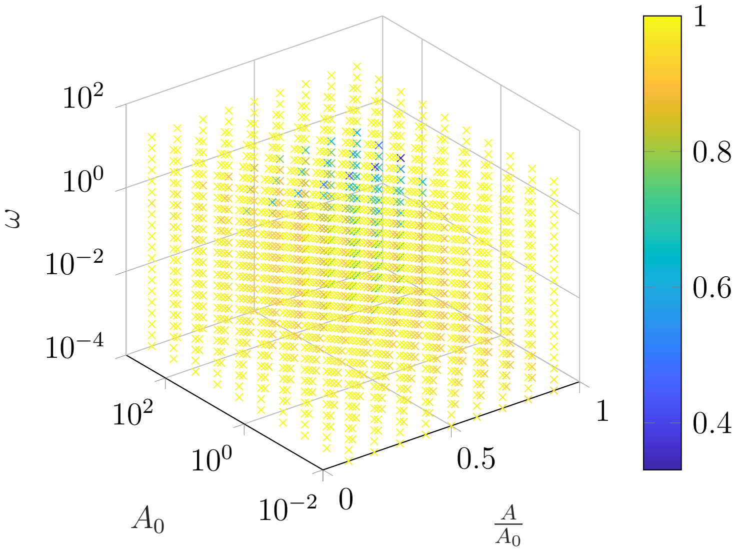

Figure 14: Proportion of basis frequency in the power spectrum of the output signal generated by the nonlinear system subject to input (22) over different parameters of the input signal.

and calculated the proportion of the basis frequency in the power spectrum, i.e.

$$p_{\text{rel}} = \frac{|c_1(\omega)|^2}{\sum_{n=1}^{10} |c_n(\omega)|^2}. \tag{26}$$

If $p_{\text{rel}} \approx 1$, this indicates that higher harmonics can be neglected. As shown in Fig. 14, this is not always the case. For large values of $\frac{A}{A_0}$ and input frequencies in the range $\omega \in [10^{-2}, 10^0]$, the value of p_{rel} drops below 0.8, suggesting that the output signal will significantly be influenced by frequency components other than the basis frequency ω. This means that the output signal will have a distorted shape.
